# The pathway to delivering injectable CAB for HIV prevention: strategies from global PrEP leaders leveraging an adapted version of the Intervention Scalability Assessment Tool (ISAT)

**DOI:** 10.1186/s43058-024-00637-1

**Published:** 2024-09-18

**Authors:** Lauren R. Violette, Kidist Zewdie, Nyawira Gitahi, Kristin Beima-Sofie, Renee Heffron

**Affiliations:** 1 Department of Epidemiology, University of Washington, Seattle, USA; 2 Department of Medicine, University of Washington, Seattle, USA; 3 Department of Global Health, University of Washington, Seattle, USA; 4 Department of Medicine, University of Alabama at Birmingham, Birmingham, USA

**Keywords:** HIV-prevention, Pre-exposure prophylaxis, Long-acting injectables, Cabotegravir, Scale-up, ISAT, Implementation, Qualitative study

## Abstract

**Background:**

Longer-acting cabotegravir (CAB) is a novel, safe, and efficacious pre-exposure prophylaxis (PrEP) for HIV prevention. As we near a time for CAB scale-up, the experience of global leaders in PrEP research and implementation can be leveraged to identify optimal strategies for scaling and integrating CAB into existing PrEP infrastructure worldwide.

**Methods:**

We recruited leaders of HIV prevention clinical trials and large PrEP programs through a combination of purposive and snowball sampling for participation in individual interviews. We conducted interviews using a semi-structured guide that compared CAB to oral PrEP and sought perspectives on barriers and strategies for CAB scale-up. Interviews were conducted virtually, audio recorded, and transcribed. We used thematic analysis, grounded in an adapted version of the Intervention Scalability Assessment Tool (ISAT), to identify critical elements for optimizing delivery of CAB.

**Results:**

From October 2021 to April 2022, we interviewed 30 participants with extensive experience in PrEP research, care, and programming. Participants worked in all seven WHO regions and reported a median of 20 years working in HIV and 10 years in PrEP. Participants agreed that CAB was efficacious and discrete, therefore having the potential to address current concerns about oral PrEP adherence and stigma. Participants indicated direct and indirect costs for provider training, expansion of existing medical infrastructure, and the current medication cost of CAB as major concerns for roll out. The true cost to the end-user and health system were unknown. There were some conflicting strategies on how to best address product targeting, presentation of efficacy, and timing of product availability with scale-up. Some thought that targeting CAB for the general population could normalize PrEP and decrease stigma, while others thought that prioritizing key populations could optimize impact by targeting those with highest risk. Overall, participants emphasized that to ensure successful CAB scale-up, communities and stakeholders must be involved at every stage of planning and implementation.

**Conclusions:**

Our evaluation found that although there is a clear and urgent need for additional HIV PrEP options beyond daily oral PrEP, CAB scale-up must be thoughtful, flexible, and based in lessons learned from oral PrEP rollout.

Contributions to the literature

- We used the Intervention Scalability Assessment Tool (ISAT), a quantitative tool used to inform decision-making for the scale-up of health interventions, as a framework for a qualitative analysis.
- To our knowledge, this is the first use of the ISAT to assess scalability of injectable cabotegravir (CAB) for HIV pre-exposure prophylaxis (PrEP).
- Our findings offer a summary of perspectives from global HIV PrEP leaders, experts, and advocates that can be used to map the process of CAB implementation using existing HIV prevention infrastructure.

## Background

Widespread delivery of oral pre-exposure prophylaxis (PrEP) for HIV prevention began in 2012 after it was found to be a safe and efficacious method of reducing HIV acquisition [1–3]. Over a decade later, the AIDS Vaccine Advocacy Coalition (AVAC) estimates that there have been almost five million PrEP initiations worldwide [4].

Despite successful scale-up across several countries and populations, there are still significant gaps in access to and uptake and sustained use of oral PrEP. Long regulatory or sourcing processes for countries, in addition to cost considerations have delayed oral PrEP scale-up in some regions [5–8]. Low product awareness or acceptability among providers or populations also contribute to decreased access [6, 8, 9]. Perceived and experienced stigma further complicate accessibility and uptake of PrEP at both the system- and individual-level [8, 10–12]. Even when available for use, oral PrEP may not be sufficiently discrete, involves frequent interaction with the healthcare system, and requires daily pill-taking to ensure efficacy [10–15].

There are several novel PrEP products in various stages of development that have the potential to address barriers to oral PrEP use, including options that have fewer side effects, are more discrete, and rely less on daily medication adherence [16–21]. Following trials demonstrating high efficacy and safety, long-acting injectable cabotegravir (CAB), a bimonthly injection, was approved for use by the United States Food and Drug Administration (FDA) in 2021 and was later recommended by the World Health Organization (WHO) in 2022 [22, 23].

Scale-up of CAB, however, is complicated by the unique challenges in delivery and programmatic integration across several regions and populations. These include, but are not limited to, insufficient or unstable funding mechanisms, overburdened health systems, and lack of product awareness [6, 19, 24–26]. As programmatic implementation of CAB approaches, it is crucial to apply lessons learned from oral PrEP to create an ideal roadmap for scale-up and ensure equitable access and delivery of CAB. As such, we conducted a qualitative evaluation to identify global PrEP leader perspectives on how to fill HIV prevention gaps and identify optimal strategies for the integration of CAB into existing PrEP infrastructure worldwide.

## Methods

Between October 5, 2021 and April 8, 2022, we recruited and conducted in-depth interviews (IDIs) with leaders of clinical trials of novel PrEP products, leaders of PrEP programs, and PrEP advocates from all seven regions of the WHO. We initially used purposive sampling to contact leaders of PrEP clinical trials and programs for interviews. These participants were identified through HIV prevention research networks, peer-reviewed publications, and scientific websites. A second wave of participants were recruited via snowball sampling through referrals made by initial participants. Recruitment continued until we reached thematic saturation. Potential participants were eligible if they were ≥ 18 years, spoke English, and had experience in biomedical HIV prevention trials or program implementation.

IDIs were conducted using semi-structured interview guides framed using the diffusion of innovation theory and explored personal experiences with research and/or implementation of programs involving oral PrEP, CAB, and other long-acting HIV PrEP methods. All IDIs were done virtually via zoom, with recorded audio (Zoom Video Communications Inc., version 5.16.2) and were later transcribed using rev.com (rev, 2022). At the time of their interview, participants also completed a brief online survey via REDCap electronic data capture tool (Vanderbilt University, version 13.10.6) to ascertain demographic and professional information, geographic reach of their work, and years of experience in both HIV and PrEP research or implementation. Interviews were conducted by LRV, KZ, NG, and RH, who identify as cisgender women living in the United States (LRV, KZ and RH) and Kenya (NG), and all have experience in PrEP- and HIV-related research and practice.

We used thematic analysis, grounded in an adapted version of the Intervention Scalability Assessment Tool (ISAT) [27] to identify critical elements for optimizing delivery of daily and long-acting PrEP products. The ISAT is an assessment tool used to assist in decision-making for health intervention funding and scale-up, particularly for high-income countries. The original tool includes a series of open-ended and Likert scale questions spanning ten domains, split into two parts, that are based on scalability and implementation considerations. We reviewed concepts captured by questions in the original ISAT tool and transformed question content into qualitative codes and code definitions to develop a preliminary codebook. We also combined two domains: delivery setting and workforce or implementation infrastructure after finding significant overlap between the concepts captured under these domains during our preliminary coding process.

A subset of transcripts were coded in Dedoose (SocioCultural Research Consultants, LLC, version 7.0.23) by members of the study team using the ISAT-informed preliminary codebook. Codes and definitions were further adapted to operationalize meaning within the HIV prevention and CAB landscape and derive a final version of the codebook. Each transcript was coded by one member of the coding team (LRV, KZ, NG, KBS, RH) using the final version of the codebook. All coded transcripts were then reviewed by another member of the team to note disagreements in application and ensure accuracy and interrater agreement. Disagreements in code application were resolved through group discussions with the entire coding team. Queries were used to generate reports by code, mapped to the appropriate ISAT domain. Queries were reviewed to identify key concepts and themes, which were summarized first by domain, and then compared between domains.

## Ethical considerations

All participants provided verbal informed consent and were reimbursed $25 USD for their participation. The University of Washington Institutional Review Board provided an exempt determination for this research.

## Results

A total of 30 global leaders in PrEP research and implementation participated in IDIs. The median age was 48.5 years (interquartile range [IQR]: 43–56) and the group was evenly split between people identifying as women and men. We did not have any participants who identified as transgender. Most participants were from Africa (47%) or the United States (27%). Professional titles included activist (*n* = 2), clinician (*n* = 6), professor (*n* = 5), program director/manager (*n* = 10), researcher (*n* = 4), and technical advisor (*n* = 3). Participants worked in all seven WHO regions and reported a median of 20 years working in HIV (IQR:15–30), and 10 years in PrEP (IQR: 6–12).

Overall, participants expressed concern for continued HIV transmission and acquisition among different populations and regions, highlighted gaps in oral PrEP access and use, and were excited about the promise of CAB to address HIV prevention needs in various settings. However, participants’ views on the implementation of CAB scale-up differed based on multiple product and contextual factors. Major concepts affecting participant beliefs about how implementation should occur are mapped to each adapted ISAT domain and described in Table 1. Key concepts affecting rationale and strategies for scaling are further detailed below.

**Table 1:** Scale-up and delivery strategies for long-acting cabotegravir for PrEP using an adaptation of the Intervention Sscalability Assessment Tool (ISAT)

Adapted ISAT Category	Operationalized Definition	Codes	Major Themes	Supporting Quote
Part A: Rationale and setting for CAB scale-up				
The problem	Describes the problem and gap in HIV prevention, who it impacts, and how the shortcomings are currently being addressed	■ HIV acquisition and transmission ■ Oral PrEP gap	■ Daily oral PrEP does not adequately address HIV prevention needs of all people and context	“We saw that in a number of people [who] were initiating oral PrEP, however, the follow-up was kind of winding down, and this was mostly because of stigma or mostly because of pill burden or people not wanting to have daily pills. So, introduction of an injectable long-acting would kind of be a solace to such populations that are [at] substantial risk and would want to prevent HIV.” -Participant #17
The intervention	Describes how CAB can address the problem	■ Benefits ■ Challenges	■ Intramuscular injection given once every two months with some concerns for drug resistance and need to attend in-person appointments ■ Good safety profile and efficacy	“I’m wondering if the initial potential attractiveness of injectable PrEP will diminish when people realize that it’s not just a shot in the arm every two months.” -Participant #20
Strategic/political context	Strategic, political, or environmental contextual factors that are potentially important influences on scale-up of CAB	■ Community and cultural ■ Political	■ Guideline development across world is ongoing and US FDA approval will encourage this further, but cost-effectiveness and demonstration studies will be crucial for product positioning and prioritization	“I think it’s farther along than any other product has been at the moment of launch […] and implementing partners are ready to deliver this product, all based on how much does the product cost and can they get access to it. So, once those two questions get answered, then we’ll be able to say whether we’re ready.” -Participant #19
Evidence of effectiveness	The level of evidence available to support the scale-up of CAB, such as scientific literature and/or other known evaluations	■ Available data ■ Missing data	■ Highly efficacious compared to daily oral PrEP in clinical trials, less concerns for adherence ■ More data are needed on use during pregnancy/breastfeeding, and among transgender women, transgender men, and non-binary persons	“For Cabotegravir I think we need those data [*during pregnancy and breastfeeding*] really, really quick and really fast before it even gets to the market so when it rolls out, it rolls out that it’s safe for everybody rather than holding some populations back and saying, ‘You know, let’s first figure this out.’” -Participant #10
Intervention cost and benefits	Consideration of the known costs of CAB implementation	■ Direct cost ■ Indirect cost	■ Direct cost is a primary concern and will be a significant barrier to product rollout ■ Indirect costs include provider training and supply chain management	“Even if they make it accessible through purchase through PEPFAR or Global Fund for low- and middle- income countries, they’re still price gouging wealthier countries. In the U.S., $22 thousand dollars a year with no generic PrEP. Who’s gonna even – why would anyone use that? So, I don’t see it as an elephant in the room. I see it as the major barrier to universal rollout of Cabotegravir.” -Participant #29
Part B: Strategies for CAB scale-up				
Fidelity and adaptation	Proposed changes to CAB implementation required or helpful for scale-up	■ Consistent ■ Inconsistent	■ Consideration of the CAB tail and how discontinuation/late doses will render users vulnerable to drug resistant HIV ■ HIV RNA testing is not feasible in many settings	“CDC guidance has come out that requires viral load or NAT testing before initiation and at every two month intervals. That’s obviously going to be very complicated –if not impossible – to implement in resource limited settings.” -Participant #11
Reach and acceptability	The likely reach and acceptability of CAB for the target population	■ Provider counseling and messaging ■ Community counseling and messaging ■ Stigma	■ Implement with population-level approach centered around product choice, led by potential end-users ■ Discrete and less product-related stigma	“And if we work with the local communities, they’ll tell us what is the best way to disseminate information and how is it going to be acceptable. It has to be socially and culturally acceptable wording and delivery” -Participant #27
Delivery setting, workforce, and implementation infrastructure	The setting within which CAB is delivered, and details implementation infrastructure required for scale-up	■ Integration into health behaviors ■ Integration into medical care ■ Social support ■ Health system ■ Subsidizing or incentivizing	■ Train healthcare workers and peers from community-based programs with the goal of normalizing PrEP use ■ De-medicalization and integration into sexual and reproductive health services will increase access but likely needs a medical setting to start	“With CAB, at least at the beginning, I don’t think we’re gonna see that differentiation in terms of the delivery of the product […] I think there is huge opportunity to differentiate the elements of the program, even if the product is all given from a particular place.” -Participant #19
Sustainability	Longer-term outcomes of the scale-up and how, once scaled up, CAB could be made sustainable over the medium to longer term	■ Sustainability	■ Ensure adequate supply chain ■ Development of newer PrEP formulations should not overshadow delivery of existing programs and products (i.e., oral PrEP)	“And then when we think about not only that cost, we think about also sustainability. That’s my biggest, biggest concern is that if we started to have long acting Cabotegravir, will we have an issue with oral PrEP being available? When you introduce many, many options, we’ve had very good experiences with ART, but you have to be careful to ensure that all of those options are available” -Participant #9

### Rationale and setting for CAB scale-up

CAB was viewed as a product that can overcome, but also exacerbate, challenges experienced with oral PrEP delivery and use. Participants noted that end-user perceived risk and actual risk are sometimes incongruent, and the longer periods of protection against HIV provided by CAB may mean populations will need to less frequently assess their own risks by not having to actively decide to take oral PrEP each day.

*“I think it’s always challenging because I don't think people are very good evaluators of their own risk. So, I think the more convenient you can make sustained prevention the less it has to be a decision tree about whether or not to continue it. […] I think Cabotegravir is a step in that right direction.”* - Participant #11; works in Africa, the Americas, Europe, and South-East Asia

Many of the study participants indicated that CAB could address issues of accessibility and adherence by reducing barriers related to stigma or fear of disclosure seen with oral PrEP. However, they also recognized that CAB may not provide additional HIV prevention coverage in contexts where adherence to oral PrEP, and therefore effectiveness of oral PrEP, is already high.

Dosing or refill frequency, which varied for oral PrEP between settings but remained constant at every two months for CAB, affected whether CAB increased or decreased the frequency with which patients would need to access the clinic or health system. Whichever product resulted in more frequent interactions with the health system was often viewed as disadvantageous. However, the decision tradeoff was further complicated by weighing increased health system interactions against relieving patients of a daily pill burden.

“*But for CAB-LA, if it is in its current form, you will then really need to dedicate into coming to the clinic every two months, which is more frequently than the visits that you need for oral PrEP because otherwise people feel, “Oh, okay. I don’t have to take pills every day. It’s easier for me, for my life” but actually coming to the clinic sometimes may be more burdensome for certain people than taking the pills every day*.” - Participant #3; works in South-East Asia

The cost of CAB was the biggest concern among all participants, regardless of geographic region. Even without knowing the exact cost of CAB, all participants believed costs would be high compared to oral PrEP and could limit implementation planning and end-user interest. Participants noted that in addition to potentially higher costs for the drug itself, there are other cost considerations including supply chain management, training providers to administer injections and educate patients, and insurance coverage coordination. Participants believed that the additional costs of CAB would be particularly burdensome in places with healthcare staffing shortages or less frequent monitoring for oral PrEP. Given cost beliefs, several participants felt that there would be less support for CAB in places with universal health coverage like the United Kingdom or Australia, especially since cost-effective alternatives like oral PrEP are available.

“*Uptake just doesn't happen in systems where the government pays for drugs until the drug is a reasonable price. So, in terms of global PrEP access, this is a huge elephant in the room.*” -Participant #22; works in the Western Pacific

Some participants expressed concern that the rollout of CAB may be slower than that of oral PrEP due to potentially higher costs, especially following a decrease in HIV prevention resources post COVID-19. However, many noted that rollout could be expedited by harnessing existing oral PrEP structures, systems, and guidelines.

“*Discussions of funding, discussions of satellite chain, discussions of training of our health workers, and incorporating all of these into our prevention guidelines. So, I’d say it shouldn’t take a long time because it will be more or less an addendum to our already existing guidelines. And the supply chain of moving to the facilities would, kind of, ride on the already existing structures.*” - Participant #17; works in Africa

In addition to cost barriers, almost all participants identified a need for additional data on CAB effectiveness and acceptability, and additional studies to identify preferences, cost-effectiveness, and real-world implementation.

“*I would see the next steps as having those demo[nstration projects] […] to me, I would still think that would be a necessary step right now to understand how it’s going to be delivered, what are the modalities, what are the strategies that can be used? And then once we are sure about that, then we roll it out and we move forward as opposed to starting to rollout and then we have to come back and start to plan all over again because we don’t have any data*.” - Participant #9; works in Africa

Participants cautioned that enthusiastic rollout of CAB without sufficient implementation and costing data could limit progress and ultimately stall successful scale-up to populations who may benefit from CAB most.

### Strategies for CAB scale-up

**Fig. 1:**
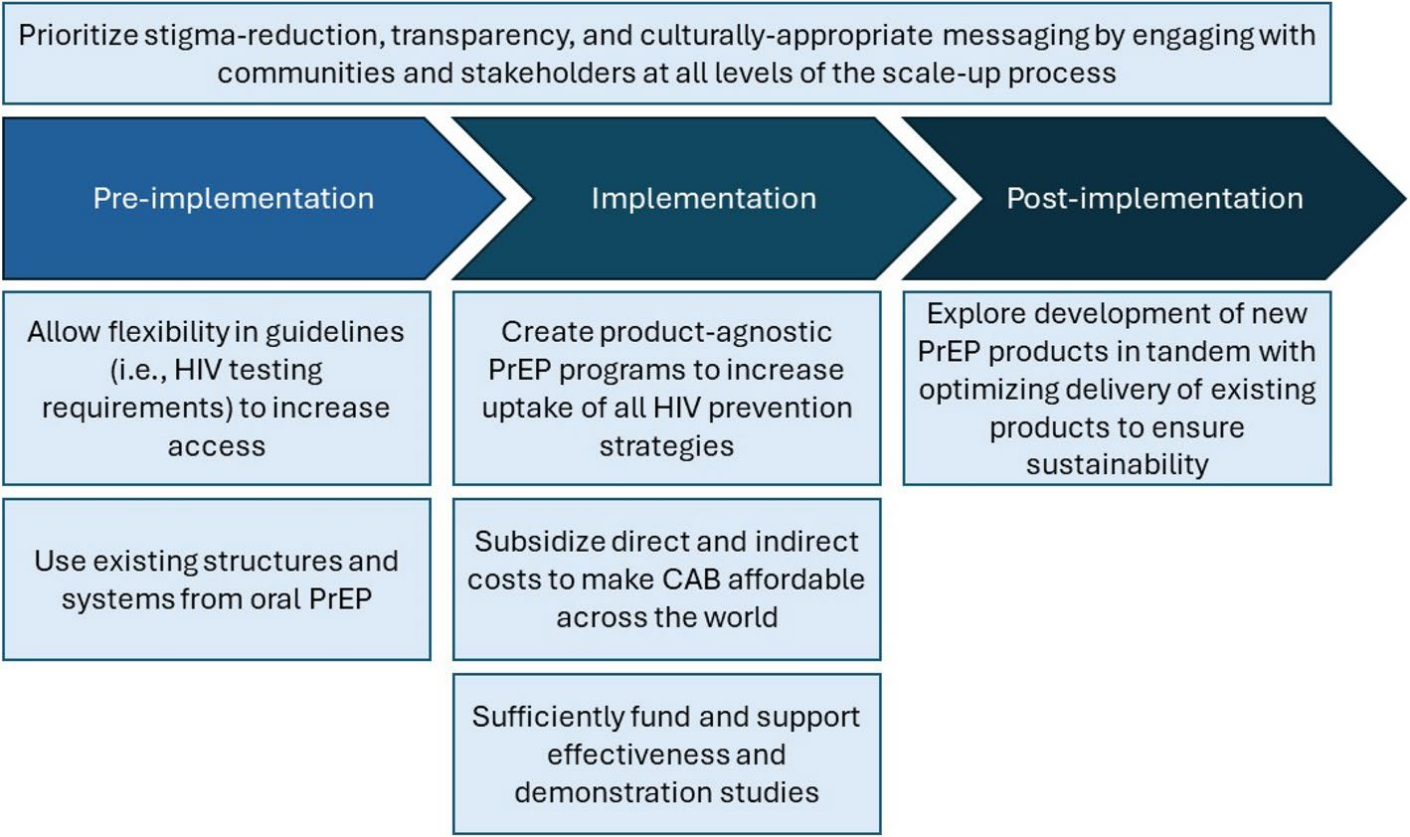
Recommendations for long-acting cabotegravir delivery

Participants suggested several strategies for CAB scale-up that focused on adaptations to delivery of the drug compared to clinical trial protocols (e.g., HIV testing requirements), types of messaging to improve reach, considerations for integration into existing health systems, and attention to sustainability during rollout. Recommendations for CAB implementation that span across all domains of the adapted ISAT framework are summarized in Fig. 1.

Participants discussed the importance of delivering CAB in a feasible way that adheres to established or likely guidelines, while also maintaining flexibility to accommodate expanded geographic and programmatic reach. For example, the current CAB guidelines in the US recommend resource-heavy RNA testing for initiation and monitoring of HIV status over time [28], but guidelines from WHO detail that RNA testing is optional but may be included by countries or programs at CAB initiation [29]. Several participants proposed using less expensive options, such as HIV self-testing or rapid testing, to better meet the resource needs of many settings with high HIV burden but low resources.

Expanding guidelines could ultimately make differentiated, community-based delivery of CAB more attainable, something most participants identified would be a key strategy for scaling CAB. The ability to quickly move to community-based delivery was thought to be easier in places already experienced with successful differentiated HIV prevention care models like in Africa.

“*Clinical services can be delivered to the community if programs are resourced to do that. I mean again I’ve worked with male circumcision programs for 15 years. You can do a circumcision in a tent under a tree, you know? Just because something needs clinical providers doesn’t mean it needs to be done in a clinic*.” – Participant #6; works in Africa, the Americas, and South-East Asia

Additionally, participants noted that taking oral PrEP to cover the tail period after CAB discontinuation, per trial protocols and currently proposed guidelines, may not be feasible to implement in practice.

“*[W]e can make a guideline that says, “You must take oral pills for two years, or a year, after taking it.” But getting clients to say, “Yeah, okay. I’ll do that. That’s a good thing,” is another question*.” – Participant #22; works in the Western Pacific

An overarching strategic area to prioritize for scale-up of CAB was stigma reduction. Product- and delivery-related stigma were commonly mentioned concerns, and participants suggested mitigation through provider and end-user created messaging and community-led delivery efforts. One of the most cited roots of stigma for oral PrEP was the fear of being seen with pills, which would be alleviated by CAB. However, CAB delivery was not viewed as stigma-free, especially if delivery were to occur within HIV or sexual health clinics. Participants suggested providing CAB in already trusted spaces within the community to reduce discrimination and stigma.

“*The benefit of CAB-LA over oral PrEP is that you won’t have a bottle of pills that looks just like a treatment bottle and the worry that if someone sees you with the bottle, you must be HIV-infected. But if someone sees you going into the clinic every two months, they’re gonna start wondering, “Why are you going to the clinic?” But it’s just different. I do think there are some benefits and stigma reduction benefits in cabotegravir as a more discreet method*” – Participant #19; works in Africa, the Americas, Europe, and South-East Asia

In addition to service location, clear and concise messaging was viewed by many participants as important for successful scale-up following several recent mis- and disinformation campaigns. Spreading scientifically accurate messages about CAB, including long-term safety metrics and information about the tail, would overcome stigma and help generate end-user demand. Participants strongly believed that to be effective, development of these messages should involve community members from key populations to ensure cultural and social relevance.

“*End users should be part of every decision made around injectable PrEP. They should influence demand creation messages. They should be part of the guidelines’ developments because they're the ones who know what will work for them and what actually suits them*.” - Participant #25; works in Africa

Although CAB was viewed as having potential to overcome barriers to daily pill-taking, there were new concerns that the less consistent routine of visiting clinic only every few months could also negatively impact adherence. Participants felt that adherence to CAB could be improved by pairing visits for CAB injections with another planned health behavior or routine that occurs on the same schedule (e.g., getting contraceptives). In addition to pairing injections with other medical activities, using social support models, including PrEP ambassadors, community care workers, and community leaders, could improve adherence. However, the delivery of social support would need to be adapted since daily and bimonthly adherence behaviors are likely different.

“*We did see a lot of adherence support groups that are there for treatment […] and that was kind of transitioned to support PrEP – oral PrEP. You know, we don’t have a lot of experience of how to support people to [come to] the clinic every two months, so this is kind of a new area, and I think we need to both adapt and build on what we did in oral PrEP, but some of that has to be new, because we haven’t done anything quite like this.”* - Participant #19; works in Africa, the Americas, Europe, and South-East Asia

There was a general consensus that development of new products and delivery of existing products must occur in tandem in order for PrEP scale-up to be successful and sustainable. Participants suggested using lessons learned from the field of contraceptives to ensure that programs providing multiple PrEP options are sustainable, while also acknowledging that HIV prevention may be more nuanced.

“*I think there’re probably some lessons from contraception. How do women learn about Depo-Provera and come back every three months? So, I think there’s some huge opportunities to build on that, but I think we have to be pretty conscious that this is not just a “get them in the clinic and inject and everything’s taken care of”.*” - Participant #19; works in Africa, the Americas, Europe, and South-East Asia

### Lack of consensus for scale-up

**Fig. 2:**
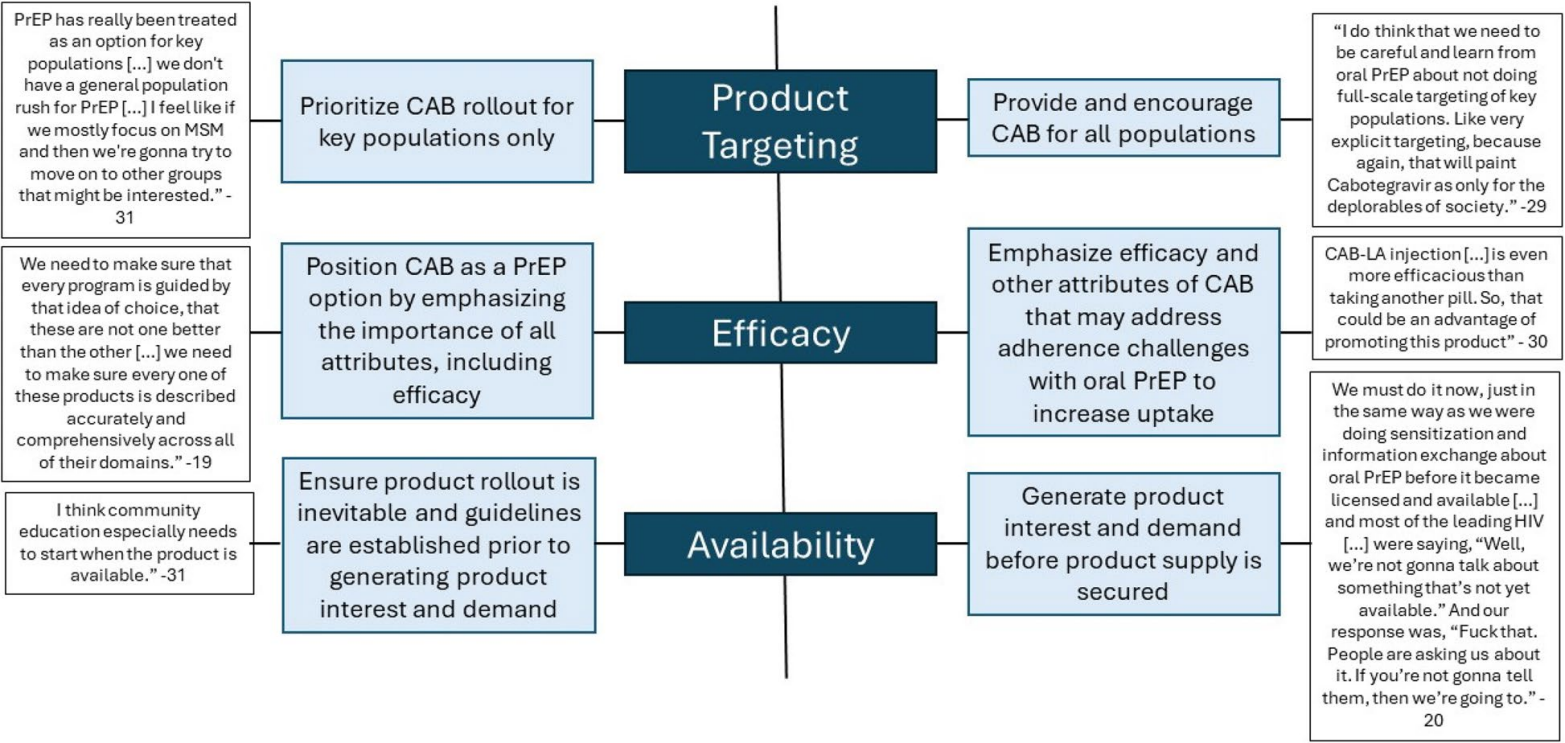
Lack of consensus for scale-up and implementation of injectable cabotegravir

Despite consensus on several strategies for scale-up, there was a lack of consensus on how to best address product targeting, efficacy, and availability (Fig. 2).

The impact of using targeted versus generalized rollout of CAB was seen as a key concern among participants that could either eliminate or drive stigma, therefore affecting product reach. From an implementation standpoint, some participants thought prioritizing CAB for key populations would be more feasible, especially since awareness of and interest in PrEP is higher among key populations compared to the general population. A more targeted rollout approach would allow for tailored messaging around risk, which could improve uptake and use. In contrast, other participants believed that targeted rollout could be harmful, especially in places where behaviors associated with increased risk of HIV acquisition are highly stigmatized or even illegal. These participants favored generalized rollout to all populations to mitigate risks and reduce stigma, therefore encouraging uptake among key populations.

As noted, the efficacy of CAB in clinical trials was high. The majority of participants cautioned that leading with the efficacy findings in presenting CAB as an option for prevention would inadvertently position it as a superior option over oral PrEP. In many settings, CAB may not prove to be superior for several reasons. Instead, participants recommended presenting CAB as an additional PrEP option by emphasizing all attributes of the method, including but not limited to, the modality, monitoring schedule, safety metrics, and efficacy. Participants argued that efficacy findings should be explained to potential end-users but with the caveat that oral PrEP, when used as instructed, has high efficacy as well. Such positioning would center end-user choice, which many strongly believe is the goal of introducing multiple PrEP options to market. Some participants, however, thought that leading with efficacy results would create more end-user demand for CAB over oral PrEP, and recommended using the high efficacy as a “selling point” for the product.

Participants were conflicted about the best strategy for ensuring product availability while simultaneously generating end-user demand for CAB. Many participants believed that demand creation and information campaigns about CAB should begin as soon as possible, even if guideline development and product rollout are yet to happen. Participants felt that social media would quickly disseminate information on CAB, and organizations should prepare for how they want to share information to combat any misinformation that could arise. Moreover, generating demand could impact how guidelines are written, and create an important opportunity for end-user involvement in the CAB implementation strategy. However, other participants recommended guideline development occur first and noted the importance of securing product supply before creating end-user demand. Their views were based on oral PrEP scale-up, where there were long lags between demand creation campaigns and product availability with the lags contributing to decreased interest in oral PrEP and prevailing uncertainty about product availability. Regardless of order, many participants did anticipate that guideline development and regulatory approvals may happen faster for CAB, as several strategies from oral PrEP regulatory processes could be adapted.

## Discussion

In this qualitative evaluation, we interviewed global PrEP leaders and stakeholders for their perspectives on how to optimize delivery of CAB through the adaptation of existing PrEP delivery systems and lessons learned from the rollout of daily oral PrEP. Overall, participants were enthusiastic about a long-acting PrEP option and believed that CAB could address barriers to oral PrEP use including dosing frequency and product-related stigma. However, participants noted several limitations of CAB and cautioned that scale-up could be stalled if issues like cost, supply chain, guideline development, and adequate community buy-in are not prioritized.

We used the ISAT framework to describe two key parts of CAB implementation described by our participants: rationale for CAB and strategies for scale-up. Participants noted several limitations of daily oral PrEP and agreed that CAB could increase accessibility to HIV prevention among populations for whom oral PrEP is not ideal. In populations or regions where PrEP adherence is sub-optimal, CAB can offer additional protection against HIV by eliminating the need to take a daily pill [17, 18]. In instances where oral PrEP adherence is high, CAB may not offer additional protection against HIV since daily pill-taking is not a challenge. Many participants stated that having multiple options for HIV prevention, however, can increase overall PrEP coverage, regardless of adherence estimates, because choice begets better usage.

It was also noted that dosing regimens for oral PrEP differ across geographic regions. In places where monthly dosing is standard, CAB offers less frequent interaction with the healthcare system. In contrast, if oral PrEP dosing is quarterly, CAB requires more interaction with the healthcare system and can introduce more burden to the end-user and PrEP programs. This difference speaks to the nuanced and location-specific approach required for understanding how CAB may fit into the current package of HIV prevention options offered to populations.

One universal concern was the cost of CAB. Cost was widely unknown at the time of interviews and posed a huge barrier to planning for rollout. Early data from real-world implementation of CAB in the US has shown that loss of cost coverage was associated with CAB discontinuation, indicating that higher end-user cost can decrease sustained use of CAB [30]. Partnerships between pharmaceutical companies and governing bodies will be crucial to bring CAB to scale, as high out-of-pocket or systems costs are likely to limit the reach of the product to populations who could benefit the most.

The second part of the ISAT detailed strategies for scale-up, mostly based on lessons learned from oral PrEP rollout. Use of existing oral PrEP infrastructure can expedite implementation of CAB, but systems will need to be adapted to include multiple HIV prevention options. In addition, guidelines need to be flexible as monitoring requirements are likely to differ between oral PrEP and CAB. For example, the country-specific recommendations for HIV RNA testing by the WHO could add burden to the health system in locations that decide to require an RNA test at CAB initiation. Even within specific regions, RNA testing may be feasible for a hospital system or clinic but could severely limit expansion of CAB delivery within community-based settings where HIV RNA testing is not as readily available. To increase access, national and local guidelines should allow for alternate forms or frequency of testing for CAB monitoring. This could include testing algorithm changes or introduction of alternative strategies such as pooled nucleic acid testing or point-of-care nucleic acid tests.

Many participants also agreed that product-agnostic programs could have the biggest population impact as offering a variety of PrEP options would ultimately cater to more people and cover a greater span of prevention needs, resulting in decreased HIV incidence. Many pointed to examples from family planning and contraceptive programs as a model for incorporating multiple prevention products into practice. As new, more preferred contraceptive options have come to market with the potential to overcome method-related reasons for non-use, contraceptive uptake and persistence has increased [31–35]. Several participants expected the same observations to hold true for HIV prevention and recent results from a randomized trial of the SEARCH (Sustainable East Africa Research in Community Health) dynamic choice HIV prevention intervention in rural Uganda and Kenya showed similar potential. Data demonstrated higher PrEP coverage among both women and men when product choice (oral PrEP, CAB, and post-exposure prophylaxis) was offered [30, 36]

Our study has several strengths. First, we recruited diverse perspectives from across the globe. Participants had a wide range of responsibilities and experience in HIV and PrEP-related research, programming, clinical care, and advocacy. This increases the representativeness of our data and makes our recommendations and strategic approach more comprehensive. All seven WHO regions were represented, providing somewhat of a global reach of our evaluation and findings. Second, we used both purposive and snowball sampling methods, which allowed us to identify key leaders and stakeholders in the field. Our evaluation used the ISAT to describe the process for bringing CAB to scale. The ISAT was developed as a quantitative tool for policy-makers to make systematic assessments of the feasibility of scale-up for different interventions in high resource settings. To date, the ISAT has been used to quantitatively assess scalability for obesity and fall prevention programs, crisis and emergency communication strategies, and mobile health interventions [37–40]. We used the ISAT to assess CAB scalability across a more broad, global scope, and did so using qualitative data. This allowed for understanding how feasible scale-up of CAB may be in regions or among populations in which limited quantitative pilot data exist. Moreover, the ISAT was selected as a framework for this analysis because it provides a comprehensive way to envision the scale-up process by including several necessary components including feasibility, acceptability, costs, sustainability, and adaptability, and provides actionable steps for policy-makers to plan for and execute implementation. With the inevitable introduction of CAB into HIV prevention programs globally, it is crucial to plan for all aspects of scale-up, instead of individual components. Our evaluation also sets a precedent for other quantitative-based implementation science frameworks to be used with qualitative data.

Our evaluation does have some limitations. First, the ISAT framework was not chosen a priori and therefore was not used in development of our interview guide. Because of this, some themes may overlap with multiple ISAT domains, and key pieces from the ISAT may be missing from our data. We did, however, adapt the ISAT framework to combine domains based on our findings when we felt significant overlap existed. Our study spanned multiple months and during that time, CAB received regulatory approval from the FDA and the WHO [28, 29]. We did not analyze data separately before and after the approvals, and it is possible that participant reactions to and suggestions for CAB scale-up may differ between periods. Finally, though our sample was representative of many different regions and professional roles, we only interviewed individuals who identified as men or women, limiting gender diversity of our sample.

## Conclusions

Novel HIV PrEP products, including CAB, are nearing the time for large-scale programmatic implementation, and there is a public health imperative to identify ways that existing oral PrEP platforms can be expanded to integrate CAB to effectively reach populations in need. Our work with global leaders in PrEP research, programming, and advocacy found that though there is a clear and urgent need for additional HIV PrEP options beyond daily oral PrEP to provide population-wide protection against HIV, CAB scale-up must be thoughtful, flexible, and based in lessons learned from oral PrEP rollout.

## Data Availability

The datasets used and/or analyzed during the current study are available from the corresponding author on reasonable request.

## Abbreviations

ISAT: Intervention Scalability Assessment Tool
CAB: Long-acting cabotegravir
CAB-LA: Long-acting cabotegravir
PrEP: Pre-exposure prophylaxis
AVAC: AIDS Vaccine Advocacy Coalition
IDI: In-depth interview
WHO: World Health Organization
USD: United States dollars
IQR: Interquartile range
RNA: Ribonucleic acid
FDA: Food and Drug Administration
